# Analysis of Hierarchical Routine Data With Covariate Missingness: Effects of Audit & Feedback on Clinicians' Prescribed Pediatric Pneumonia Care in Kenyan Hospitals

**DOI:** 10.3389/fpubh.2019.00198

**Published:** 2019-07-16

**Authors:** Susan Gachau, Nelson Owuor, Edmund Njeru Njagi, Philip Ayieko, Mike English

**Affiliations:** ^1^Health Services Unit, Kenya Medical Research Institute-Wellcome Trust Research Programme, Nairobi, Kenya; ^2^School of Mathematics, University of Nairobi, Nairobi, Kenya; ^3^Department of Non-Communicable Disease Epidemiology, London School of Hygiene and Tropical Medicine, London, United Kingdom; ^4^Department of Infectious Disease Epidemiology, London School of Hygiene and Tropical Medicine, London, United Kingdom; ^5^Nuffield Department of Medicine, University of Oxford, Oxford, United Kingdom

**Keywords:** missing data, multiple imputation, PAQC score, routine data, audit and feedback, pediatrics

## Abstract

**Background:** Routine clinical data are widely used in many countries to monitor quality of care. A limitation of routine data is missing information which occurs due to lack of documentation of care processes by health care providers, poor record keeping, or limited health care technology at facility level. Our objective was to address missing covariates while properly accounting for hierarchical structure in routine pediatric pneumonia care.

**Methods:** We analyzed routine data collected during a cluster randomized trial to investigating the effect of audit and feedback (A&F) over time on inpatient pneumonia care among children admitted in 12 Kenyan hospitals between March and November 2016. Six hospitals in the intervention arm received enhance A&F on classification and treatment of pneumonia cases in addition to a standard A&F report on general inpatient pediatric care. The remaining six in control arm received standard A&F alone. We derived and analyzed a composite outcome known as Pediatric Admission Quality of Care (PAQC) score. In our analysis, we adjusted for patients, clinician and hospital level factors. Missing data occurred in patient and clinician level variables. We did multiple imputation of missing covariates within the joint model imputation framework. We fitted proportion odds random effects model and generalized estimating equation (GEE) models to the data before and after multilevel multiple imputation.

**Results:** Overall, 2,299 children aged 2 to 59 months were admitted with childhood pneumonia in 12 hospitals during the trial period. 2,127 (92%) of the children (level 1) were admitted by 378 clinicians across the 12 hospitals. Enhanced A&F led to improved inpatient pediatric pneumonia care over time compared to standard A&F. Female clinicians and hospitals with low admission workload were associated with higher uptake of the new pneumonia guidelines during the trial period. In both random effects and marginal model, parameter estimates were biased and inefficient under complete case analysis.

**Conclusions:** Enhanced A&F improved the uptake of WHO recommended pediatric pneumonia guidelines over time compared to standard audit and feedback. When imputing missing data, it is important to account for the hierarchical structure to ensure compatibility with analysis models of interest to alleviate bias.

## Introduction

Routine data are widely used in many countries to monitor quality of care and to inform intervention programmes for better patients' health outcomes (1).

Routine data can also be used to highlight areas of concern in clinical performance thus prompting actions and strategies to improve practice at individual or institutional levels (2). Prior studies show that quality of care vary across place and time in spite of standard clinical guidelines (3). These variations can be attributed to multiple factors including changes in clinical guidelines, degree of task complexity, and patient's characteristics, clinician characteristics in addition to organizational and contextual factors at hospital level (3–5). Between 2013 and 2014, the Kenya Medical Research Institute-Wellcome Trust Research Programme in collaboration with the Ministry of Health, the Kenya Pediatric Association and 14 county-level hospitals initiated a partnership known as the Clinical Information Network (CIN). The main aim of CIN is to collect and use routine pediatric data to promote adoption and adherence to recommended clinical practices through audit and feedback (A&F) cycles (3, 5–7). While such data from multiple sites enhance generalization of results to wider population, it leads to complex hierarchical data structures, for instance, patients clustered within clinicians, who are then clustered within hospitals.

Besides complex structures, routine data are subject to missing information at any level of hierarchy. Missing information may occur due to lack of documentation of care processes by health care providers, poor record keeping, or limited health care technology at facility level (1, 8, 9). In the occurrence of missing data, appropriate missing data methods at analysis stage are recommended to avoid biased results (10) informing clinical policies and ultimately leading to poor patients care and outcomes (11).

In the recent past, there has been an increase in literature on quality of care among children admitted with common childhood illnesses in low and middle income countries (3, 12–15). However, majority of the studies account for variation at patient and hospital levels ignoring variation due to clinicians characteristics in spite of their critical role in delivery of routine care (16). Besides, missing data is a common problem across these studies. Majority of the studies report using complete case analysis (13, 17, 18) and multiple imputation (15, 19, 20). A major limitation of complete case records is biased and inefficient parameter estimates due to information loss. In studies where multiple imputation is used to handle missing data, the nature and details of the imputation model are rarely reported posing uncertainty about conclusions and barriers for replicate analyzes. Furthermore, when missing data occur in multilevel data context, incompatibility between the imputation model and the analysis models potentially leads to biased estimates, underestimated cluster level variances, and overestimated individual level variances (10, 21–23). For example, incompatibilities occur when the imputation model assumes data are single level (i.e., ignoring multilevel structure) while the analysis model of interest is multilevel.

In this study, we aim to address missing covariates while properly accounting for hierarchical structure in inpatient routine data set, that is, patients nested within clinicians who are then nested within hospitals. Specifically, we analyze data from a cluster randomized trial investigating the effect of enhanced audit and feedback on clinicians' prescribed pediatric pneumonia care in Kenyan hospitals. To achieve this objective, we construct and analyze pneumonia Pediatric Admission Quality of Care (PAQC) score adapted to new WHO recommendations on assessment and treatment of inpatient pediatric pneumonia cases. PAQC score is a newly developed ordered composite measure used to benchmark quality of care among children admitted with common childhood illnesses in low and middle income settings.

The remainder of this paper is structured as follows: In the Methods section we present a description of pneumonia trial data followed by statistical analysis methods for cluster correlated and missing data methods, respectively. Thereafter, we present results before and after multiple imputation and conclude with a discussion.

## Methods

### Study Design

In this study we analyzed data from a cluster randomized trial conducted by KEMRI-Wellcome Trust Research programme between March 2016 and November 2016. Details of the trial and the study population are described in full elsewhere (5, 24). In summary, the trial was embedded within the larger CIN study (ongoing) (6, 7, 25). The primary goal of the trial was to investigate whether enhanced audit and feedback improved quality of inpatient pediatrics pneumonia care (i.e., assessment, diagnosis, and treatment of childhood pneumonia) in Kenyan hospitals following new pneumonia guidelines recommended by the World Health Organization (WHO) in 2013 (26). Six hospitals were randomized to receive a standard audit and feedback report on general inpatient pediatric care (control arm). The remaining six hospitals received a standard audit and feedback report in addition to an enhanced audit and feedback targeting assessment, classification and treatment of pneumonia cases (intervention arm) (5, 24). Trained data clerks abstracted routine data from the medical records into Research Electronic Data Capture (REDCap) tool after patient's discharge from general pediatric wards. Data abstraction process was guided by a standard operational procedure manual (5). Patients' data spanned history of illness, physical examination, diagnosis, laboratory investigations, treatments, and discharge plans (5, 24). Details of admitting clinician including sex and professional qualification were also recorded into a separate database linked to the patients' database by a unique clinician code.

Data quality assurance (DQA) exercises were conducted by CIN research assistants in each hospital every 3 months to check consistencies with data clerk's entries. The Kenya Ministry of Health and Kenya Medical Research Institute's Scientific and Ethical Review Unit approved data collection without individual patient's consent (5).

### Outcome: Pneumonia Pediatric Admission Quality of Care Score

Our outcome of interest was pneumonia PAQC score adapted to 2013 WHO pediatric pneumonia treatment guidelines. As earlier mentioned, PAQC score is a summary measure spanning three quality of care domains namely, assessment, clinical diagnosis, and treatment of common childhood illnesses including pneumonia, malaria, diarrhea, and dehydration. Details on PAQC score construction and validation are described in full elsewhere (12, 27). With regard to pneumonia PAQC, there are three binary subcomponents in the assessment domain. The first subcomponent represents assessment and documentation of two primary signs and symptoms required for pneumonia identification (i.e., presence of cough or difficulty in breathing). The value 1 in the binary indicator denotes documentation of both cough and difficulty in breathing as either present or absent while 0 denotes lack of documentation of least one primary sign and symptom in a patient's medical record.

The second binary indicator represents assessment and documentation of secondary signs and symptoms required for pneumonia severity classification (i.e., chest indrawing, respiratory rate, grunting, central cyanosis, oxygen saturation, ability to drink, or altered level of alertness). The value 1 in the binary indicator denotes documentation of all secondary signs and symptoms, respectively, while 0 denotes lack of documentation of least one secondary signs and symptom. The third binary indicator of the assessment domain corresponds to 1 when primary and secondary pneumonia signs and symptoms (all primary and secondary signs and symptoms combined) are documented and 0 otherwise (26).

The second PAQC score domain entails integration of information on presenting signs and symptoms by admitting clinician to correctly diagnose and classify pneumonia severity (i.e., severe pneumonia or pneumonia). For example, pneumonia was the correct diagnosis for a child who, in addition to cough and/or difficult breathing (primary signs), presented with lower chest indrawing or respiratory rate >50 for patients aged 2–11 months (or respiratory rate <40 for patients aged 12–59 months) in the absence of all other secondary signs and symptoms. In this study, a binary indicator was created with value 1 representing correct pneumonia severity classification (i.e., is, pneumonia severity documented in the medical record by the admitting clinician was in line with severity implied by presenting signs and symptoms) and 0 representing misclassified pneumonia severity.

The third PAQC score domain consists of two binary indicators. The first binary variable indicates whether oral amoxicillin was prescribed for pneumonia cases (denoted by 1) or not (denoted by 0). The second binary variable indicates whether oral amoxicillin was prescribed according to guideline recommended doses (26). In order to determine correctness of the dose, we created a new variable “dose per kilo body weight” using actual dose given at point of care, patient's weight, and frequency of administration. Among pediatric pneumonia cases, the recommended oral amoxicillin dose should range between 32 and 48 international units per kilogram *(IU/Kg)* every 12 h. The new variable was then transformed into a binary variable with 1 representing correct dose (that is, dose per kilo body weight and frequencies of administration are in line with guidelines recommendations) and 0 representing incorrect dose (incorrect in either dose per kilo body weight or frequency of administration) or missing dose. Subsequently, we summed all the six binary components across domains to obtain PAQC score; an ordinal outcome on a 7-point scale. We constructed pneumonia PAQC score at patient level. A minimum score of zero corresponded to inappropriate pneumonia care and maximum score of six represented complete adherence to new pneumonia guidelines across domains of care. To assess performance in terms of adherence to pediatric pneumonia guidelines during the trial period, we calculated and plotted the LOESS smoothing curves and the corresponding 95% confidence bands for the mean monthly PAQC score for each intervention arm.

### Covariates

The covariates of interest were intervention arm, follow up time in months with their interaction, hospital malaria prevalence status, and hospital admission workload. At clinician level, gender, and cadre were considered (here cadre refers to clinician's level of training that is, clinical officers with diploma-level training and medical officers with a bachelor's degree level training). At patient level, we considered sex, number of comorbid illnesses, and age at admission. Prior to analysis, we converted age for all the patients into months before categorizing them into two age groups that is, patients aged 2–11 months and patients aged 12–59 months. With regard to comorbidities, we determined the total number of clinical diagnoses documented in patient's medical records. The diagnoses of interest included malaria, malnutrition, HIV, Asthma, Tuberculosis (TB), rickets, anemia, diarrhea, and dehydration. For each patient, we created separate binary variables for the diagnosis above with value 1 denoting the presence of a disease and 0 denoting absence of a disease. We then summed the binary indicators and categorized patients into four groups, that is those with 0, 1, 2, 3 or more comorbidities, respectively.

### Missing Data Concepts

In the analysis of partially observed data, assumptions were made about the missingness mechanism generating the data (10). Suppose *Y* (representing both response and independent variables) is an *N* × *p* matrix denoting a hypothetical data set containing p variables (*j* = 1,…,p) for the *i*th study subject, (*i* = 1,2,3,…,N). For each study subject, *Y*_*i*_ can be partitioned into observed and missing components denoted by ${Y_{i}}^{obs}$ and ${Y_{i}}^{miss}$, respectively. Further letting a missingness indicator *R*_*i*_ take the value 1 if *Y*_*i*_ is observed and 0 if *Y*_*i*_ is missing. Then according to Rubin (28) data are said to be missing completely at random (MCAR) when the probability of missing values in variable is independent of the variable itself or any other observed variable in the data set that is, $P\left(R_{i}|{Y_{i}}^{miss},\;{{\text{Y}}_{i}}^{obs}\right)\;=P\left(R_{i}\right)$. When the probability of missing values in a variable does not depend on the variable of interest but are conditionally dependent on other observed variables in the data set, then data are said to be missing at random (MAR) and denoted by $P\left(R_{i}|{Y_{i}}^{miss},\;{{\text{Y}}_{i}}^{obs}\right)\;=P\left(R_{i}|{Y_{i}}^{obs}\right)$. When MAR assumption does not hold, then data are said to be Missing Not at Random (MNAR). MNAR mechanism occurs when the missingness depends on the actual value of the missed observation (10).

### Investigating the Missing Data Mechanism

Before analyzing partially observed data, it was important to investigate plausible missing data mechanisms (10, 29). In this study we generated binary missingness indicators (*R*_*i*_) for partially observed variables in the pneumonia trial data set. The binary missingness indicators were analyzed separately using a logistic regression model below

(1) $$\begin{array}{r} logit\left[P\left(R_{i}\right)\right]\;=X_{i}\beta \end{array}$$

where X_*i*_ is a vector of fully observed variables for the ith subject. The vector β denotes fixed regression parameters to be estimated. When the probability of missingness is independent on fully observed variables (*P*-values for the regression coefficients > 0.05), a variable is said to be MCAR. On the other hand, when the probability of missingness is dependent on fully observed variables (*P*-values for the regression coefficients <0.05), then MAR assumptions holds and restricting analysis to complete observations yields bias and inefficient estimates (10, 29, 30). Similarly, when the probability of missingness is dependent on fully observed covariates but independent of the response variable, then covariate dependent MAR assumptions holds and restricting analysis to complete observations yields unbiased but inefficient estimates due to information loss (10, 29, 30). We also used graphical methods to investigate missing data patterns underlying pneumonia trial data (Figure A1 in Supplementary Material).

### Multiple Imputation

Multiple imputation (MI) involves substituting each missing value with a set of plausible values given the observed data and an imputation model (10, 31). MI is commonly used assuming a MAR mechanism but can also be used when data are MNAR. Multiple imputed data sets are then analyzed using standard methods and results pooled into a single inference using Rubin's Rule (32). Multiple imputation is preferred over other missing data methods such as list wise or pairwise deletion because uncertainty about the missing values is taken into account (10, 23, 30, 31, 33). Additionally, MI separates imputation phase from analysis phase therefore allowing inclusion of auxiliary variables in the imputation model that are predictive of missing variables and the missingness mechanism (10, 23, 27, 33–35).

In this study, we imputed missing level 1 and level 2 variables within the joint modeling framework where replacement values are drawn from a multivariate normal distribution in a single step. Multilevel MI was implemented in the newly developed *jomo and mitmil* packages in R (version 3.4.3) which allows imputation of categorical variables with more than two levels in the second and higher levels of the multilevel structure (36). For the *i*th patient nested within *j*th clinician in hospital *l*, we defined a two level JM imputation model corresponding to

(2) $$\begin{array}{l} Y_{i,j,l}^{\left(1\right)}=X_{i,j,l}^{\left(1\right)}\beta^{\left(1\right)}+b_{j,l}^{\left(1\right)}+e_{i,j,l}^{\left(1\right)}\\ Y_{j,l}^{\left(2\right)}=X_{j,l}^{\left(2\right)}\beta^{\left(2\right)}+b_{j,l}^{\left(2\right)}\\ e_{i,j,l}\sim N\left(0,\sigma_{e}^{2}\right),\;and\;\left(b_{j,l}^{\left(1\right)},b_{j,l}^{\left(2\right)}\right)\sim N\left(0,\Sigma_{b}\right) \end{array}$$

where ${Y_{i,j,l}}^{\left(1\right)}$ and ${Y_{j,l}}^{\left(2\right)}$ are vectors of partially observed level 1 variables (patient's sex) and level 2 variables (clinician's sex and cadre), respectively. Predictor variables (${X_{i,j,l}}^{\left(1\right)}$) of missing patient's sex included fully observed follow-up time interacted with feedback arm, hospital admission workload and hospital malaria prevalence status, patient's PAQC score, patient's age and number of comorbid illnesses. Level 2 predictors (${X_{j,l}}^{\left(2\right)}$) for missing clinicians' sex and cadre included follow-up time interacted with feedback arm, hospital admission workload, and hospital malaria prevalence status. Column vectors β^1^ and β^2^ denote level 1 and level 2 fixed effects, respectively. A clinician random intercept (*b*_*j, l*_) was included to account for clustering at clinicians' level and to ensure compatibility with substantive models of interests. A burn-in of 1,000 updates and a 1,000 iterations between each of the 30 imputations were considered. We used trace plots to assess convergence (37). Final estimates were pooled according Rubin's rules.

### Statistical Analysis

We considered two model families to analyze pneumonia trial data, that is, generalized estimating equations (GEE) and random effects models. The random effects and GEE models differ in terms of estimation and interpretation of parameter estimates (30). We considered both models in order to assess the stability of inferences and conclusions within and across the two methods before and after multiple imputation.

#### Generalized Estimating Equations (GEE) Model

Generalized estimating equations (GEE) proposed by Liang and Zeger (38) is a quasi-likelihood method for modeling correlated responses within the marginal (population averaged) family of models (29, 30). In GEE model a working correlation structure is adopted. However, the parameter estimates in GEE model are consistent even when the association structure is misspecified (29, 39). A GEE model is given by

(3) $$\begin{array}{r} h^{-1}\left\{E\left(Y_{i}|X_{i}\right)\right\}=X_{i}\beta \end{array}$$

where the link function *h*^−1^(•) is a known function, *X*_*i*_ is a design matrix for the fixed effects and β is the vector of unknown regression parameters. The vector of regression parameters is interpreted in terms of average response over the population rather than prediction of the effect of changing covariates on a given study subject (29).

When the responses are ordered and the proportional odds assumptions of parallel logits hold, the cumulative logits (proportional odds) model is considered (40). For instance, considering ordered pneumonia PAQC score (outcome) for the *i*th patient nested within *j*th clinician in hospital *l*, the proportional odds GEE model of interest corresponds to

(4) $$\begin{array}{l} logit\left[P\left(Y_{PACQ\;Score:\;i,j,l}\leq k\right)\right]=\alpha_{k}+\beta_{1}X_{age\;group:\;i,j,l}\\ +\beta_{2}X_{patient^{\prime }s\;sex:\;i,j,l}\;+\beta_{3}X_{comobidity:i,j,l}+\beta_{4}X_{clinician^{\prime }s\;cadre:j,l}\\ +\beta_{5}X_{clinician^{\prime }s\;sex:\;j,l}+\beta_{6}X_{admission\;workload:\;l}\\ +\beta_{7}X_{malaria\;prevalence:\;l}+\beta_{8}X_{time\;in\;months:l}*X_{trial\;arm:l} \end{array}$$

where α_*k*_, *k* = 1,2,3,4,5,6 are PAQC score intercepts and β′*s* are regression coefficients common across all *k*−1 cumulative logits.

#### Random Effects Model

In contrast to population-averaged models, random effects models are useful when drawing inferences with respect to the subject-specific parameters. Given the covariates and random effects, the responses are assumed to be conditionally independent in this model (29, 30). A random effects model is denoted by

(5) $$\begin{array}{r} h^{-1}\left\{E\left(Y_{i}|X_{i}\right)\right\}\;=X_{i}\beta +Z_{i}b_{i}\\ {\;b}_{i}\sim N\left(0,\Sigma \right) \end{array}$$

where *h*^−1^(•) is a known link function, *X*_*i*_ and *Z*_*i*_ are design matrices for the fixed effects and random effects while β and *b*_*i*_ are vectors of fixed and random parameters, respectively. The vector *b*_*i*_is assumed to be sampled from a multivariate normal distribution with mean vector **0** and covariance matrix Σ. The vector of regression parameters (β) has subject specific interpretation in terms of the transformed mean response for in individual. Considering pneumonia trial data with ordinal PAQC score as above, proportional odds random intercepts model of interest corresponds to

(6) $$\begin{array}{l} logit\left[P\left(Y_{PACQ\;Score:\;i,j,l}\leq k\right)\right]\\ =\alpha_{k}+\beta_{1}X_{age\;group:\;i,j,l}+\beta_{2}X_{patient^{\prime }s\;sex:\;i,j,l}\\ +\beta_{3}X_{comobidity:i,j,l}+\beta_{4}X_{clinician^{\prime }s\;cadre:j,l}+\beta_{5}X_{clinician^{\prime }s\;sex:\;j,l}\\ +\beta_{6}X_{admission\;workload:\;l}+\beta_{7}X_{malaria\;prevalence:\;l}\\ +\beta_{8}X_{time\;in\;months:l}*X_{trial\;arm:l}+b_{jl} \end{array}$$

where α_*k*_, *k* = 1,2,3,4,5,6 are PAQC score specific intercepts, β′*s* are estimated regression coefficients (common across all *k*−1 cumulative logits) and *b*_*j, l*_ are clinician's random intercepts. Hospital level random effects were not considered in these analyses due to the few number of clusters.

#### Statistical Tests for Multiple Parameters

We used Wald tests and likelihood-ratio tests to determine covariates with statistically significant effect on pneumonia PAQC score. The likelihood-ratio tests was used to test for statistical significance of covariates in the random effects models (10, 41, 42). On the other hand, Wald tests suggested by Rubin (10, 41) was used for the GEE model. The full (saturated) models contained all the covariates while the reduced (null) models dropped one covariate at a time. The tests were conducted on complete case records and after multiple imputation. Details on multi-parameter hypothesis tests after MI using Wald tests and likelihood-ratio tests are available in Carpenter and Kenward (10, p. 53–54) and Van Buuren (42, p. 157–158). All analyses were conducted in R version 3.4.3. A 5% level of significance was considered under complete case analysis and after MI of missing covariates.

## Results

### Descriptive Summaries

In total, 2,299 children aged 2–59 months were admitted in general pediatric wards with childhood pneumonia in 12 CIN hospitals during the trial period. We linked patients and clinicians' databases using unique clinician code present in both databases with a success rate of 92.5% (2,127/2,299) after exclusion of 172/2,299 case records lacking admitting clinician's information. This resulted to three levels of clustering i.e., 2,127 patients admitted by 378 clinicians in 12 hospitals. Of the 2,127 pneumonia cases, 953/2,127 (44.8%) were admitted in six hospitals assigned to enhanced A&F (intervention) arm. The number of pneumonia cases varied across hospitals with a range of 42–356 patients (Table 1).

**Table 1:** Descriptive characteristics of hospitals, clinicians and patients in pneumonia trial data.

	**H1**	**H2**	**H3**	**H4**	**H5**	**H6**	**H7**	**H8**	**H9**	**H10**	**H11**	**H12**	**Total**
Enhanced A&F arm	No	Yes	No	No	Yes	Yes	Yes	Yes	No	No	No	Yes	
Admission workload	Low	Low	High	Low	Low	High	Low	Low	Low	High	High	Low	
Malaria prevalence	High	Low	High	Low	Low	Low	High	High	Low	Low	Low	High	
Pneumonia admissions, *n* (%)	132 (6.21)	215 (10.11)	210 (9.87)	243 (11.42)	110 (5.17)	356 (16.74)	63 (2.96)	167 (7.85)	88 (4.14)	172 (8.09)	329 (15.57)	42 (1.97)	2,127 (100)
Patients aged 2–11 months, *n* (%)	44 (33.3)	79 (36.7)	71 (33.8)	89 (36.6)	49 (44.6)	193 (54.5)	22 (34.9)	70 (41.9)	45 (51.1)	99 (57.6)	129 (39.2)	13 (30.95)	903 (42.5)
Patients aged 12–59 months, *n* (%)	88 (66.7)	136 (63.3)	139 (66.2)	154 (63.4)	61 (55.5)	162 (45.5)	41 (65.1)	97 (58.1)	43 (48.9)	73 (42.4)	200 (60.8)	29 (69.1)	1,224 (57.5)
Male patients, *n* (%)	80 (60.6)	118 (54.9)	103 (49.1)	138 (56.8)	55 (50.0)	194 (54.5)	35 (55.6)	100 (59.9)	42 (47.7)	95 (55.2)	181 (55.1)	23 (54.8)	1,164 (54.72)
Female patients, *n* (%)	52 (39.4)	97 (45.1)	107 (50.9)	101 (41.6)	55 (50.0)	162 (45.5)	27 (42.9)	67 (40.1)	46 (52.3)	76 (44.2)	141 (42.9)	19 (45.2)	950 (44.6)
Missing patients sex, *n* (%)	0 (0.0)	0 (0.0)	0 (0.0)	4 (1.7)	0 (0.0)	0 (0.0)	1 (1.6)	0 (0.0)	0 (0.0)	1 (0.6)	7 (2.1)	0 (0.0)	13 (0.6)
0 comorbidities, *n* (%)	29 (21.9)	121 (56.3)	30 (14.3)	155 (63.8)	55 (50.0)	219 (61.5)	24 (38.1)	52 (31.1)	30 (34.1)	70 (40.7)	191 (58.1)	19 (45.2)	995 (46.8)
1 comorbidity, *n* (%)	64 (48.5)	56 (26.1)	109 (51.9)	41 (16.9)	22 (20.0)	62 (17.4)	23 (36.5)	65 (38.9)	36 (40.9)	51 (29.7)	87 (26.4)	17 (40.5)	633 (29.8)
2 comorbidities, *n* (%)	28 (21.2)	31 (14.4)	54 (25.7)	41 (16.9)	22 (21.6)	63 (17.7)	12 (19.1)	36 (21.6)	14 (15.9)	37 (21.5)	37 (11.3)	6 (14.3)	381 (17.9)
3 ≥ comorbidities, *n* (%)	11 (8.3)	7 (3.3)	17 (8.1)	6 (2.5)	11 (10.0)	12 (3.4)	4 (6.4)	14 (8.4)	8 (9.1)	14 (8.1)	14 (4.3)	0 (0.0)	118 (5.5)
Number of clinicians: *n* (%)	31	36	43	33	25	36	24	39	32	20	44	15	378
Female clinicians, *n* (%)	15 (54.55)	11 (30.56)	15 (34.9)	13 (39.4)	2 (8.0)	14 (38.9)	13 (54.2)	16 (41.0)	0 (0.0)	0 (0.0)	24 (54.6)	5 (33.3)	128 (33.9)
Male clinicians, *n* (%)	16 (45.45)	18 (50.0)	28 (65.2)	20 (60.6)	8 (32.0)	10 (27.8)	11 (45.8)	23 (59.0)	3 (9.4)	1 (5.0)	20 (45.4)	10 (66.7)	168 (44.4)
Clinicians with missing sex, *n* (%)	0 (0.0)	7 (19.4)	0 (0.0)	0 (0.0)	15 (60.0)	12 (33.3)	0 (0.0)	0 (0.0)	29 (90.6)	19 (95.0)	0 (0.0)	0 (0.0)	82 (21.7)
Clinicians' cadre: *CO, n* (%)	0 (0.0)	0 (0.0)	0 (0.0)	2 (6.1)	3 (12.0)	0 (0.0)	0 (0.0)	0 (0.0)	1 (3.1)	0 (0.0)	0 (0.0)	0 (0.0)	6 (1.6)
Clinicians' cadre: *CO* interns, *n* (%)	20 (64.5)	18 (50.0)	31 (72.1)	20 (60.6)	2 (8.0)	14 (38.9)	16 (66.7)	29 (74.4)	1 (3.1)	0 (0.0)	25 (56.82)	8 (53.3)	184 (48.7)
Clinicians' cadre: *MO*^‡^, *n* (%)	1 (3.2)	12.8 (2.8)	0 (0.0)	0 (0.0)	0 (0.0)	1 (2.8)	0 (0.0)	1 (2.6)	0 (0.0)	1 (5.0)	1 (2.3)	0 (0.0)	6 (1.6)
Clinicians' cadre: *MO* interns, *n* (%)	10 (32.3)	10 (27.8)	12 (27.9)	11 (33.3)	5 (20.0)	9 (25.0)	7 (29.2)	9 (23.1)	1 (3.1)	0 (0.0)	18 (40.9)	7 (46.7)	99 (26.2)
Clinicians with missing cadre, *n* (%)	0 (0.0)	7 (19.4)	0 (0.0)	0 (0.0)	15 (60.0)	12 (33.3)	1 (4.1)	0 (0.0)	29 (90.6)	19 (95.0)	0 (0.0)	0 (0.0)	83 (21.9)

‡ *CO-Clinical Officer, MO-Medical Officer, H1–H12 denote hospitals participating in the trial*.

Five out of 12 hospitals were drawn from high malaria endemic regions (three control and two intervention hospitals) while the remaining seven hospitals (four control and three intervention hospitals) were drawn from low malaria regions in Kenya (25). Furthermore, four in 12 hospitals were high admission workload hospitals that is, more than 1,000 pediatric admissions per annum (three control and one intervention hospitals) while 8/12 were low admission workload hospitals i.e., <1,000 pediatric admissions per annum (three control and five intervention hospitals) irrespective of admission diagnosis. On average, there were 32 clinicians per hospital with a standard deviation of nine clinicians. The number of patients per clinician ranged between 3 and 46. Majority of the admitting clinicians were clinical officer interns at 48.7% (185/378) followed by Medical officer interns at 26.2% (99/378). Clinical officer and medical officers accounted for 1.6% (6/378) each. Approximately, 21.9% (83/378) and 21.7% (82/378) clinicians had missing gender and cadre, respectively (Table 1). In subsequent analyses we grouped clinicians into two cadres from the initial four. That is, clinical officers (CO) combining clinical officers and clinical officer interns and medical officers (MO) combining medical officers and medical officer interns, respectively. Approximately, 42% (903/2,127) of patients were aged between 2 and 11 months and 45% (950/2,127) were females. Patient's sex was missing in 0.7% (17/2,127) of case records (Table 1).

**Figure 1:**
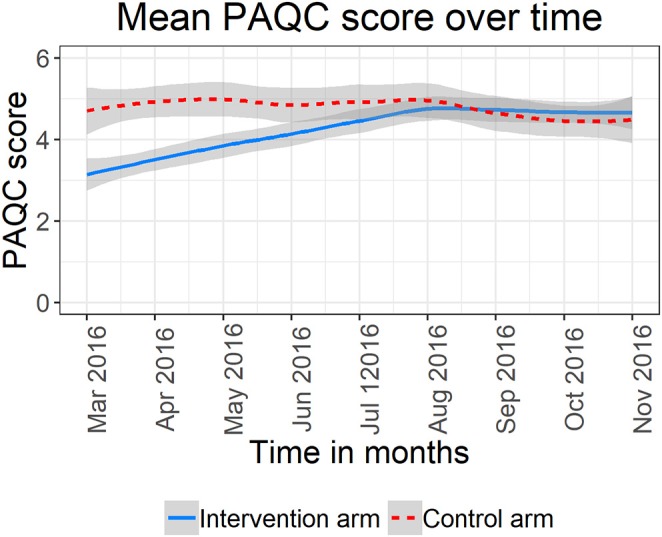
Mean PAQC score and 95% confidence bands for six hospitals in the standard A&F arm and six hospitals in the enhance A&F arm.

Examining pneumonia PAQC score over time graphically, hospitals in the standard A&F arm (red curve) exhibited a higher mean PAQC score at baseline with no significant fluctuations over time (Figure 1). On the other hand, hospitals assigned to enhanced A&F arm (blue curve) had a lower mean PAQC score at baseline which rapidly improved toward higher score in the first 6 months of follow-up. Although enhanced A&F arm's trend line surpassed that of standard A&F arm after 6 months of follow-up, the 95% confidence bands of the two intervention arms overlapped substantially (Figure 1).

**Figure 2:**
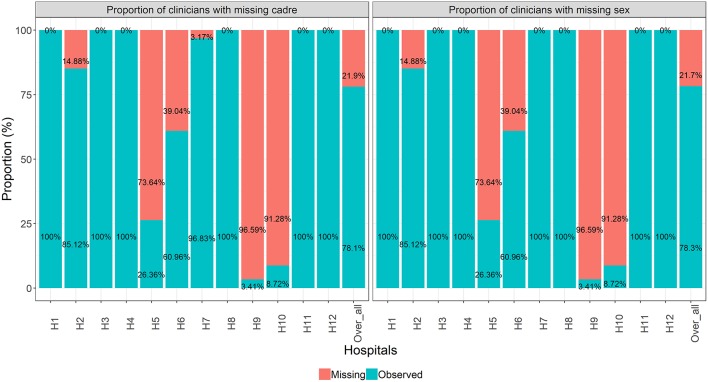
Proportion of missing clinicians' cadre and sex at hospital level and across all hospitals combined.

An assessment of missing data patterns suggested a multivariate missing data pattern (Figure A1 in Supplementary Material). The missing data pattern further revealed similarities between of missing clinician's cadre and sex. That is, nearly all clinicians with missing sex had missing cadre as well. Further investigations into missing data patterns showed that missing clinicians' cadre and sex only occurred in six out of 12 hospitals (Figure 2).

Logistic regression results on plausible mechanisms underlying pneumonia trial data indicated that the probability of missing patient's sex was neither dependent on the outcome (PAQC score) nor fully observed covariates (interaction between intervention arm and follow up time in months, hospital admission workload, and malaria prevalence, patient's age group, and the number of presenting comorbid illnesses). That is, the *P*-values were >0.05 suggesting a MCAR mechanism (Table A1 in Supplementary Material). On the other hand, the probabilities of missing clinician's cadre and gender were dependent on both the outcome and fully observed covariates suggesting evidence against MCAR (Table A1 in Supplementary Material). Therefore, we imputed missing data assuming a MAR mechanism. MI diagnostic test indicated satisfactory convergence (Figure A2 in Supplementary Material).

### Random Effects and GEE Model Results

Test for proportional odds assumption was not statistically significant at 5% level (*P* = 0.17). Therefore, we assumed parallel logits and fitted proportional odds models to complete case records and imputed datasets. In Table 2, we present the likelihood ratio test and Wald test results for proportional odds random effects and GEE model, respectively. After MI of missing covariates, we observed consistent results between the random effects model and the GEE model in terms of statistical significance of covariates of interest (Table 2). Specifically, we found statistically significant interaction effect between intervention arm and follow-up time. Similarly, admission workload at hospital level was significant at 5% level. At patients' level, age and the number of comorbidities were statistically significant while at clinicians' level, sex showed significant effect on pneumonia PAQC score (Table 2).

**Table 2:** Likelihood ratio test and Wald test statistics for random effects model and GEE model under complete case analysis and after multilevel multiple imputation of missing covariates.

	**Random effects model**				**GEE model**			
	**Complete case analysis** ***N*** **=** **1,619 (76.1%)**		**Multilevel MI** ***N*** **=** **2,127 (100%)**		**Complete case analysis** ***N*** **=** **1,619 (76.1%)**		**Multilevel MI** ***N*** **=** **2,127 (100%)**	
**Effect**	**LRT**	***P*-value**	**LRT**	***P*-value**	**Wald test**	***P*-value**	**Wald test**	***P*-value**
Patients' age	3.49	0.06	4.66	0.03	4.18	0.04	7.81	0.01
Patients' sex	0.08	0.77	0.01	0.92	0.003	0.96	0.02	0.88
Comorbidities	4.46	0.02	4.83	0.03	2.42	0.49	5.48	0.02
Clinicians' sex	5.06	0.02	4.02	0.04	6.32	0.01	4.47	0.03
Clinicians' cadre	0.01	0.91	0.23	0.63	1.36	0.24	2.96	0.08
Hospital workload	0.143	0.71	3.39	0.04	1.46	0.23	4.95	0.03
Malaria prevalence	0.067	0.79	1.35	0.25	0.98	0.32	0.012	0.91
Time (months)	11.98	< 0.001	14.16	< 0.001	11.37	0.003	11.16	< 0.001
Enhanced A&F arm	28.58	< 0.001	17.51	< 0.001	28.86	< 0.001	17.76	< 0.001
Time × Enhanced A&F	14.92	0.02	14.16	< 0.001	17.85	< 0.001	9.45	< 0.001

*LRT, Likelihood ratio test; A&F, Audit and feedback; MI, Multiple imputation; GEE, Generalized estimating equations*.

In Table 3, we present proportional odds ratios and the corresponding 95% confidence interval obtained after fitting the random intercepts model and GEE models before and after multilevel multiple imputation. Standard errors before and after MI are presented in Table A2 (Supplementary Material). For the GEE model, we reported robust (empirically corrected) standard errors which were in agreement with model based (naive) standard errors (Table A2 in Supplementary Material). Under complete case analysis, only 1,619/2,127 (76.1%) case records were considered.

**Table 3:** Odds ratios (95% confidence intervals) estimated under complete case analysis and after multilevel multiple imputation of missing covariates.

	**Random effects model**				**GEE model**			
	**Complete case analysis** ***N*** **=** **1,619 (76.1%)**		**Multilevel MI** ***N*** **=** **2,127 (100%)**		**Complete case analysis** ***N*** **=** **1,619 (76.1%)**		**Multilevel MI** ***N*** **=** **2,127 (100%)**	
**Effect**	**Odds ratios** **(95% CI)**	***P*-value**	**Odds ratios** **(95% CI)**	***P*-value**	**Odds ratios** **(95% CI)**	***P*- value**	**Odds ratios** **(95% CI)**	***P*-value**
Intercept: PAQC score 0	Reference		Reference		Reference		Reference	
Intercept: PAQC score 1	0.06 (0.031, 0.14)	< 0.001	0.18 (0.071, 0.375)	< 0.001	6.13 (3.188, 9.456)	< 0.001	4.18 (2.010, 6.128)	< 0.001
Intercept: PAQC score 2	0.07 (0.036, 0.138)	< 0.001	0.17 (0.082, 0.365)	< 0.001	7.73 (3.258, 18.316)	< 0.001	4.98 (2.056, 2.078)	< 0.001
Intercept: PAQC score 3	0.22 (0.11, 0.42)	0.03	0.53 (0.251, 1.105)	0.07	3.12 (1.345, 7.23)	0.03	2.02 (0.852, 4.809)	0.08
Intercept: PAQC score 4	0.67 (0.342, 1.294)	0.12	1.63 (0.779, 3.427)	0.67	1.29 (0.561, 2.981)	0.56	0.84 (0.354, 1.987)	0.96
Intercept: PAQC score 5	2.74 (1.401, 5.347)	< 0.001	6.69 (3.166, 14.14)	< 0.001	0.44 (0.192, 1.012)	0.12	0.29 (0.122, 0.678)	< 0.001
Intercept: PAQC score 6	7.24 (3.678, 4.253)	< 0.001	7.79 (8.336, 3.964)	< 0.001	0.21 (0.089, 0.501)	< 0.001	0.14 (0.057, 0.336)	< 0.001
Age-group: 12–59	1.20 (0.991, 1.464)	0.06	1.19 (0.986, 1.454)	0.09	1.15 (0.922, 1.432)	0.09	1.16 (0.932, 1.454)	0.08
Patients' sex: males	0.97 (0.806, 1.174)	0.77	0.97 (0.805, 1.173)	0.93	0.95 (0.759, 1.185)	0.96	0.95 (0.760, 1.183)	0.91
Comorbidities: 1	0.99 (0.783, 1.267)	0.94	0.99 (0.782, 1.253)	0.93	1.02 (0.810, 1.295)	0.84	1.03 (0.815, 1.304)	0.80
Comorbidities: 2	1.01 (0.766, 1.327)	0.95	1.01 (0.767, 1.326)	0.96	1.01 (0.779, 1.304)	0.94	1.01 (0.781, 1.312)	0.92
Comorbidities: ≥3	0.63 (0.398, 0.985)	0.04	0.61 (0.387, 0.955)	0.03	1.37 (0.906, 2.063)	0.14	1.41 (0.937, 2.126)	0.09
Clinicians' sex: female	1.51 (1.057, 2.183)	0.02	1.53 (1.064, 2.195)	0.02	1.44 (1.095, 1.910)	0.01	1.45 (1.106, 1.894)	0.01
Clinicians' cadre: MO	1.02 (0.709, 1.468)	0.91	1.04 (0.720, 1.49)	0.98	1.18 (0.878, 1.582)	0.24	1.20 (0.888, 1.611)	0.18
Hospital workload: low	0.93 (0.624, 1.376)	0.71	1.12 (1.080, 1.372)	0.04	1.42 (0.974, 2.068)	0.23	1.40 (1.103, 2.063)	0.02
Malaria prevalence: low	0.95 (0.644, 1.401)	0.79	0.94 (0.640, 1.389)	0.25	1.18 (0.748, 1.865)	0.32	1.18 (0.742, 1.87)	0.95
Time (months)	1.05 (0.969, 1.145)	0.22	1.05 (0.967, 1.141)	0.81	0.99 (0.904, 1.094)	0.86	0.99 (0.905, 1.103)	0.40
Enhanced A&F arm	0.18 (0.095, 0.349)	< 0.001	0.18 (0.093, 0.341)	< 0.001	0.11 (0.054, 0.227)	< 0.001	0.11 (0.053, 0.236)	< 0.001
Time × Enhanced A&F	1.15 (1.018, 1.307)	0.02	1.16 (1.020, 1.308)	< 0.001	1.27 (1.125, 1.484)	< 0.001	1.29 (1.117, 1.482)	< 0.001
Variance (standard error) between random clinicians' intercepts	1.328 (1.151)		1.161 (1.073)					

*SE, Standard Error; CI, Confidence interval; MO, Medical Officer; A&F, Audit and feedback*.

This loss information led to larger standard errors comparison to those obtained after MI of missing covariates in both random effects and GEE model families. Furthermore, the proportional odds ratios were consistently smaller under complete case analyses compared to those obtained after MI (Table 3). These results were an indication of bias and inefficiency of parameters estimated under complete case analysis. The six PAQC score intercepts presented in Table 3 denote thresholds (cut points) differentiating adjacent levels of the response variable. For example, intercept 1 in Table 3 denote the odds of PAQC score = 1 vs. PAQC score ≥ 2 for a female patient aged 2–11 months admitted with no comorbidities admitted by a male medical officer in a high workload hospitals located in high malaria prevalence region. The individual fixed effect parameters are the proportional odds ratios of individual variables on PAQC score holding all other variables in the model constant.

From study results, enhanced audit and feedback led to improve uptake of new pneumonia pediatric guideline over time. For instance, considering a patient admitted in an intervention hospital (enhanced audit and feedback arm), the odds of PAQC score = 1 vs. PAQC score ≥ 2 were 1.16 (95% CI: 1.02–1.308) times higher the odds of a patients admitted in a control hospital, for a unit increase in follow-up time and holding other variables at reference levels. Likewise, for a patient admitted in an intervention hospital, the odds of PAQC score = 1 vs. PAQC score ≥ 2 were 1.29 (95% CI: 1.17–1.482) times higher the odds of a patients admitted in a control hospital, for a unit increase in follow-up month (GEE model after MI). These interpretations hold for all other response (PAQC score) levels.

The study results also exhibited shifts in statistical significance before and after multiple imputation for selected variable. Specifically, adjusting for other variables, complete cases analysis lead to insignificant difference between low and high admission workload hospitals on levels of PAQC score in both random effects model and GEE model where the 95% CI confidence intervals contained the value 1. But after MI, the odds of higher pneumonia PAQC score in low workload hospitals were 1.12 (95% CI: 1.08–1.372) and 1.40 (95% CI: 1.103–2.063) times higher than for high workload hospitals for the random intercepts and GEE model, respectively (Table 3).

With regard to random effects model, the variance component between clinicians and the corresponding standard error were inflated under complete cases analysis. A possible explanation for this results is that clinicians with missing cadre and sex were discarded under complete case analysis resulting to fewer number of clinicians (clusters) hence inflated clinicians' variability. On the other hand, all clinicians were retained after MI hence lower variability between clinicians.

## Discussion

This study sought to investigate the effect of enhanced A&F on routine pediatric pneumonia care in 12 Kenyan hospitals during a cluster randomized trial. In the analysis we adjusted for patients, clinicians, and hospital levels factors while accounting for covariate missingness across the three levels of hierarchy. The number of pneumonia admissions varied widely across hospitals during the trial period. The outcome of interest (pneumonia PAQC score) is a composite measure representing multiple aspects of pediatric pneumonia care on a 7-point ordinal scale. The advantage of using composite outcomes over individual performance measures is increased statistical efficiency (43–47). Although we reported and analyzed a fully observed outcome, we note that variations in pneumonia PAQC on the 7-point ordinal scale was attributable to missing data in some of the subcomponents in addition to inappropriate pneumonia care across domains of care (12). Specifically, missing components and those corresponding to inappropriate care were scored zero. Among covariates, clinician variables exhibited the highest proportions of missingness. Approximately 21% of all admitting clinicians had missing sex and cadre, respectively. These observations were consistent with previous results of a cluster randomized trial evaluating the effectiveness of a multifaceted intervention to improve admission pediatric care in eight Kenyan hospitals (10, 48). In the said study, 14 and 20% of the clinicians had missing sex and years of experience, respectively.

In contrast, patient level variables were fully observed except patient's sex which had <1% missingness. The sharp contrast missingness between clinicians and patients level variables could be due the fact that continued CIN audit and feedback reports focus on the documentation of patient level variables rather than documentation of clinicians' characteristics. Through preliminary investigations, we established that missing clinicians' characteristics occurred in six out of 12 hospitals participating in the trial. The patterns of missingness in the two clinicians level variables was highly correlated. That is, clinicians who did not document their sex were also likely not to document their cadre and vice versa.

To alleviate bias and inefficiency, we used multiple imputation within the joint modeling (JM) imputation framework assuming a MAR mechanism (10, 30, 31). Although JM imputation framework does not address the full range of complexities that are typical of multilevel data (22, 23), it was preferred due to its flexibility coupled with recent statistical software developments in handling categorical variables with more than two levels in second and higher levels of hierarchy (36).

Consistent with our expectations, results demonstrated that multilevel imputation led to more precise parameter estimates compared to complete case analyses in both random effects and GEE models. Adjusting for patients, clinicians and hospital level factors, enhanced A&F improved uptake and adherence to recommended pediatric pneumonia guidelines over time among children aged 2–59 months admitted in six CIN hospitals during the trial period compared to standard A&F on general inpatient pediatric care. The significant difference in the uptake of the pneumonia guidelines between the intervention arms could be due to difference in baseline performance observed in the Loess curves. That is, control hospitals exhibited high baseline performance (on average) thus leaving smaller room for improvement compared to low baseline performance in the enhanced A&F arm hence larger room for improvement over time. These results were consistent with those of the primary analysis (24).

A key difference between our study and that primary analysis is that whereas we analyzed a composite outcome spanning three quality of care domain, Ayieko et al. (24) considered proportion of patients with correct pneumonia classification and treatment, respectively. Furthermore, our study accounted for clinicians' characteristics in addition to patients and hospitals level characteristics accounted for in the primary analysis. From results, the quality of pneumonia care differed between male and female clinicians. It was also evident that junior clinicians (medical officers and clinical officer interns) were responsible for much care during the trial period. However, the quality of care provided did not differ between the cadres. The high number of interns is an indication that hospitals in the trial were teaching and referral hospitals.

### Strengths and Implications of the Study

In this study, we investigated plausible missing data mechanism underlying pneumonia trial data. Though often ignored, this step is important in assessing and understanding the implications of missingness in a given data set under analysis. That is, inefficient estimates or both biased and inefficient estimates. In addition to missing data mechanism, we evaluated missing data patterns underlying the trial data set. This was useful in revealing trends and gaps in the quality of routine care. Insight into such information is useful when designing cost effective follow-up or new interventions programmes for optimal and efficient utilization of already stretched resources (49). For instance, based on our study results, a follow up intervention programme aimed at improving documentation and reporting of clinician characteristics should be directed to specific hospitals low documentation of clinicians' level data while resources in hospitals with good documentation practices should be directed elsewhere.

To address missing data, we employed recent statistical software tools to impute missing variables in routine pediatric data. Our choice of imputation tools and method was in consideration of the hierarchical structure of the data and type of variables in the data set. This ensured compatibility between imputation and analysis models of interest thus minimizing bias in parameter estimates (10, 23). Further, our choice of proportion odds models to analyze the ordinal outcome was ascertained through formal test further enhancing the validity of our study results. In instances when the proportional odds assumptions are violated, multinomial logistic regression model is recommended (40). In contrast to previous studies reporting quality of inpatient pediatric routine care in CIN hospitals (3, 13, 15), our study accounted for clinicians who are essential for the delivery of health intervention (16). Ignoring variation at clinician level may lead to biased estimates, overestimation or underestimation of variations in other levels of clustering (50).

## Limitations

A limitation of this study is that we relied on data collected after patient discharge. Therefore, we are unable to ascertain if patients received pneumonia care as documented by health workers (24). We imputed missing data assuming MAR mechanism. Therefore, sensitivity analyses will be undertaken to explore the robustness of the inferences to MAR assumptions.

## Conclusion

Adjusting for hospitals, admitting clinicians, and patient level factors, enhanced audit, and feedback improved uptake of WHO recommended pediatric pneumonia guidelines compared to standard audit and feedback. Additionally, female clinicians and hospitals with low admission workload were associated with higher uptake of the new pediatric pneumonia guidelines during the trial period. In both random effects and marginal model, parameter estimates were biased and inefficient under complete case analysis. Therefore, multiple imputation is recommended. When analyzing partially observed data with more than one level of clustering, it is paramount to accounts for the hierarchical structure in the imputation model to ensure compatibility with analysis models of interest and hence alleviate bias.

## Ethics Statement

The Kenya Ministry of Health and Kenya Medical Research Institute's Scientific and Ethical Review Unit approved the use of de-identified patient data obtained through retrospective review of medical records without individual patient consent.

## Author Contributions

SG conducted the analyses. Feedback on the analytic approach was provided by EN, NO, PA, and ME. SG drafted the initial manuscript with feedback on subsequent drafts provided by all authors who then approved the final manuscript.

### Conflict of Interest Statement

The authors declare that the research was conducted in the absence of any commercial or financial relationships that could be construed as a potential conflict of interest. The handling Editor declared a shared affiliation, though no other collaboration, with several of the authors EN and PA, within the last two years.
