# Supplementary material for: Analysis of Hierarchical Routine Data With Covariate Missingness: Effects of Audit & Feedback on Clinicians' Prescribed Pediatric Pneumonia Care in Kenyan Hospitals
Source: Front Public Health. 2019 Jul 16;7:198. doi: 10.3389/fpubh.2019.00198 (PMC6646705; doi:10.3389/fpubh.2019.00198)
Supplement: Supplementary file 1 [file Data_Sheet_1.docx]

Table A1: Multiple logistic regression model parameter estimates (standard errors) for the probabilities of missing patients’ sex, clinician’s sex and cadre.

|  | Patient’s sex | | Clinician’s sex | | Clinician’s cadre | |
| --- | --- | --- | --- | --- | --- | --- |
| Variable | Estimate (s.e) | P value | Estimate (s.e) | P value | Estimate (s.e) | P value |
| Paqc score 1 | 0.66 (0.86) | 0.99 | 9.36(0.001) | <0.001 | 9.35 (0.01) | <0.001 |
| Paqc score 2 | -2.02 (0.83) | 0.97 | -8.42(0.001) | <0.001 | -8.42 (0.01) | <0.001 |
| Paqc score 3 | -2.16 (0.62) | 0.85 | 6.44(0.002) | 0.002 | 6.44 (0.01) | <0.001 |
| Paqc score 4 | 0.33 (0.36) | 0.78 | -3.98(0.001) | 0.003 | -3.98 (0.01) | 0.002 |
| Paqc score 5 | 0.43 (0.16) | 0.67 | 1.81(0.001) | 0.002 | 1.81 (0.01) | 0.001 |
| Paqc score 6 | 1.12 (0.50) | 0.69 | -0.26(0.001) | 0.03 | -0.26 (0.01) | 0.02 |
| Age-group:12-59 months | -0.56(0.63) | 0.38 | 0.21(0.96) | 0.03 | -0.56(0.63) | 0.04 |
| Comorbidity 0 | 0.91 (0.82) | 0.12 | -1.96(0.97) | 0.04 | -1.96 (0.97) | 0.02 |
| Comorbidity 1 | 2.04 (6.4) | 0.99 | -2.09(1.15) | 0.06 | -2.09 (1.14) | 0.21 |
| Comorbidity 2 | 2.04 (3.7) | 0.98 | -2.03(0.002) | 0.10 | -2.03 (0.01) | 0.11 |
| Malaria prevalence: low | -9.5 (6.24) | 0.96 | -10.13(0.001) | 0.01 | -10.13 (0.01) | 0.01 |
| Hospital workload: Low | 1.63(0.39) | 0.68 | -1.06(1.08) | 0.03 | -1.06 (1.08) | 0.02 |
| Enhanced A&F arm | 1.47 (2.34) | 0.43 | 0.21(0.001) | 0.03 | 0.21 (0.01) | <0.001 |
| Time (months) | -0.34 (0.13) | 0.07 | -0.48(0.002) | 0.03 | -0.47 (0.01) | 0.04 |
| Time*Enhanced A&F arm | 0.26 (0.40) | 0.68 | -0.05(0.001) | <0.001 | -0.06 (0.001) | <0.001 |

Table A2: Standard errors estimated in random effects model and GEE model under complete case analysis and after multilevel multiple imputation.

|  | **Random effects model** | | **GEE Model** | |
| --- | --- | --- | --- | --- |
|  | **Complete case analysis N=1619 (76.1%)** | **Multilevel MI**  **N=2127 (100%)** | **Complete case analysis N=1619 (76.1%)** | **Multilevel MI**  **N=2127 (100%)** |
| Effect | Standard error | Standard error | Standard error | Standard error |
| Intercept: PAQC score 0 | ref | ref | ref | ref |
| Intercept: PAQC score 1 | 1.231 | 1.074 | 1.031 | 1.010 |
| Intercept: PAQC score 2 | 1.075 | 0.381 | 0.332 | 0.332 |
| Intercept: PAQC score 3 | 0.383 | 0.378 | 0.329 | 0.330 |
| Intercept: PAQC score 4 | 0.380 | 0.378 | 0.336 | 0.336 |
| Intercept: PAQC score 5 | 0.380 | 0.382 | 0.334 | 0.334 |
| Intercept: PAQC score 6 | 0.384 | 0.387 | 0.342 | 0.341 |
| Age-group:12-59 | 0.389 | 0.093 | 0.09 | 0.086 |
| Child sex: Males | 0.100 | 0.096 | 0.084 | 0.084 |
| Comorbidities: 1 | 0.121 | 0.120 | 0.122 | 0.120 |
| Comorbidities :2 | 0.141 | 0.140 | 0.131 | 0.130 |
| Comorbidities: ≥ 3 | 0.231 | 0.230 | 0.209 | 0.208 |
| Clinician sex: female | 0.186 | 0.185 | 0.183 | 0.182 |
| Clinician Cadre: MO | 0.186 | 0.185 | 0.166 | 0.163 |
| Hospital workload: low | 0.202 | 0.201 | 0.178 | 0.160 |
| Malaria prevalence: low | 0.198 | 0.198 | 0.190 | 0.191 |
| Time (months) | 0.042 | 0.042 | 0.038 | 0.038 |
| Enhanced A&F arm | 0.332 | 0.332 | 0.335 | 0.338 |
| Time× Enhanced A&F | -.064 | 0.063 | 0.060 | 0.060 |


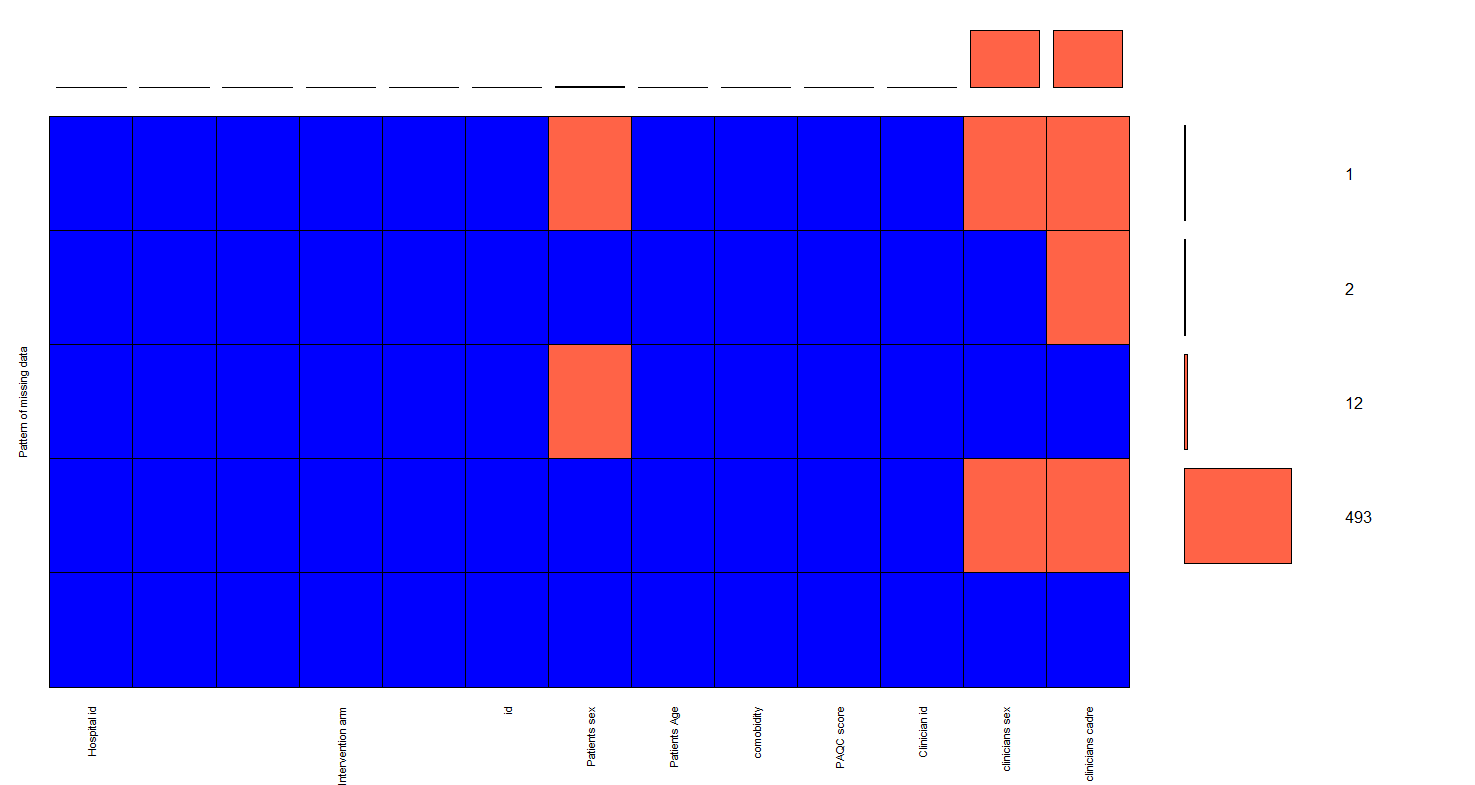


**Figure A1: Missing data pattern underlying pneumonia trial data**

Figure A2: Example of a chain portraying satisfactory multilevel multiple imputation convergence
